# Sensorimotor Inhibition and Mobility in Genetic Subgroups of Parkinson's Disease

**DOI:** 10.3389/fneur.2020.00893

**Published:** 2020-09-04

**Authors:** Douglas N. Martini, Rosie Morris, Valerie E. Kelly, Amie Hiller, Kathryn A. Chung, Shu-Ching Hu, Cyrus P. Zabetian, John Oakley, Kathleen Poston, Ignacio F. Mata, Karen L. Edwards, Jodi A. Lapidus, Thomas J. Grabowski, Thomas J. Montine, Joseph F. Quinn, Fay Horak

**Affiliations:** ^1^Department of Neurology, Oregon Health and Science University, Portland, OR, United States; ^2^Department of Rehabilitation Medicine, University of Washington School of Medicine, Seattle, WA, United States; ^3^Portland Veterans Affairs Health Care System, Portland, OR, United States; ^4^Department of Neurology, University of Washington School of Medicine, Seattle, WA, United States; ^5^Veterans Affairs Puget Sound Health Care System, Seattle, WA, United States; ^6^Department of Neurology and Neurological Sciences, Stanford School of Medicine, Palo Alto, CA, United States; ^7^Lerner Research Institute, Genomic Medicine, Cleveland Clinic Foundation, Cleveland, OH, United States; ^8^Department of Epidemiology, University of California, Irvine, Irvine, CA, United States; ^9^Biostatistics & Design Program, Oregon Health and Science University, Portland, OR, United States; ^10^Department of Radiology, University of Washington School of Medicine, Seattle, WA, United States; ^11^Department of Pathology, Stanford University School of Medicine, Palo Alto, CA, United States

**Keywords:** short-latency afferent inhibition, SAI, postural sway, balance, gait, *GBA*, *APOE*

## Abstract

**Background:** Mobility and sensorimotor inhibition impairments are heterogeneous in Parkinson's disease (PD). Genetics may contribute to this heterogeneity since the apolipoprotein (*APOE*) ε4 allele and glucocerebrosidase (*GBA*) gene variants have been related to mobility impairments in otherwise healthy older adult (OA) and PD cohorts. The purpose of this study is to determine if *APOE* or *GBA* genetic status affects sensorimotor inhibition and whether the relationship between sensorimotor inhibition and mobility differs in genetic sub-groups of PD.

**Methods:** Ninety-three participants with idiopathic PD (53 non-carriers; 23 ε4 carriers; 17 *GBA* variants) and 72 OA (45 non-carriers; 27 ε4 carriers) had sensorimotor inhibition characterized by short-latency afferent inhibition. Mobility was assessed in four gait domains (pace/turning, rhythm, trunk, variability) and two postural sway domains (area/jerkiness and velocity) using inertial sensors.

**Results:** Sensorimotor inhibition was worse in the PD than OA group, with no effect of genetic status. Gait pace/turning was slower and variability was higher (*p* < 0.01) in PD compared to OA. Postural sway area/jerkiness (*p* < 0.01) and velocity (*p* < 0.01) were also worse in the PD than OA group. Genetic status was not significantly related to any gait or postural sway domain. Sensorimotor inhibition was significantly correlated with gait variability (*r* = 0.27; *p* = 0.02) and trunk movement (*r* = 0.23; *p* = 0.045) in the PD group. In PD non-carriers, sensorimotor inhibition related to variability (*r* = 0.35; *p* = 0.010) and trunk movement (*r* = 0.31; *p* = 0.025). In the PD ε4 group, sensorimotor inhibition only related to rhythm (*r* = 0.47; *p* = 0.024), while sensorimotor inhibition related to pace/turning (*r* = −0.49; *p* = 0.046) and rhythm (*r* = 0.59; *p* = 0.013) in the PD *GBA* group. Sensorimotor inhibition was significantly correlated with gait pace/turning (*r* = −0.27; *p* = 0.04) in the OA group. There was no relationship between sensorimotor inhibition and postural sway.

**Conclusion:** ε4 and *GBA* genetic status did not affect sensorimotor inhibition or mobility impairments in this PD cohort. However, worse sensorimotor inhibition was associated with gait variability in PD non-carriers, but with gait rhythm in PD ε4 carriers and with gait rhythm and pace in PD with *GBA* variants. Impaired sensorimotor inhibition had a larger effect on mobility in people with PD than OA and affected different domains of mobility depending on genetic status.

## Introduction

Mobility impairment is intrinsically linked to Parkinson's disease (PD). The severity of mobility impairment in PD is heterogeneous, suggesting that underlying factors that play a role in mobility impairment. In addition to basal ganglia dysfunction, people with PD exhibit reduced cortical sensorimotor inhibition as assessed by short-latency afferent inhibition (SAI) with transcranial magnetic stimulation (TMS) (1–3). However, these studies reveal inconsistencies regarding the impact of disrupted sensorimotor inhibition on mobility disability in people with PD.

Sensorimotor inhibition is associated with decreased gait speed in people with PD (1), increased dual-task cost on stride length in PD fallers (2), and increased gait variability in people with PD (Martini et al., under review at J Gerontol: Med Sci), though not with freezing of gait (4). Within genetic subgroups of people with PD, neither a leucine-rich repeat kinase 2 mutation nor a *Parkin* gene mutation was associated with worse SAI (5–7). Importantly, none of these investigations assessed relationships among genetic status, SAI, and mobility. A potential reason for heterogeneity of mobility disability and of sensorimotor inhibition in PD could be the presence of the apolipoprotein (*APOE*) ε4 allele or glucocerebrosidase (*GBA*) gene variants (8, 9). The disparity in which specific domains are affected could be tied to genetic differences within PD to which specific gait or postural sway variables are characterized. Assessing relatively independent domains of gait or postural sway constructed by combinations of individual gait measures could help provide a more complete picture of gait performance (10).

The *APOE* ε4 allele has been associated with accelerated decline in gait speed in healthy, older men (11). Since PD is twice as likely to occur in men (12), it is pertinent to look at the effect of the ε4 allele status on gait and postural sway in people with PD, and men with PD, in particular. People with Alzheimer's disease, a disease inherently linked to the ε4 allele and known to be associated with cholinergic insufficiency, have worse SAI than people without Alzheimer's and cholinergic medication improves SAI, suggesting that acetylcholine loss may contribute to poor SAI (13, 14). To date, no attempt has been made to assess the link between ε4 allele carrier status and gait dysfunction and their relationships to sensorimotor inhibition in people with PD. Similarly, variants of the *GBA* gene are also associated with accelerated mobility decline in the elderly and with decline in the clinical postural instability and gait disorder phenotype in people with PD, regardless of sex (15–17). However, no investigation has approached sensorimotor inhibition in *GBA* carriers with PD and related SAI heterogeneity to their mobility performance.

The purpose of this study was to determine if ε4 carrier or *GBA* variant status is associated with reduced sensorimotor inhibition in people with PD and whether worse sensorimotor inhibition is associated with worse gait and postural sway performance. We hypothesized that sensorimotor inhibition and mobility will be worse in ε4 carriers, especially those with PD, and also in those with PD and *GBA* variants. Further, we hypothesize that decreased gait pace and increased gait variability will be related to worse sensorimotor inhibition.

## Materials and Methods

This study was approved by Institutional Review Boards at both University of Washington and Oregon Health & Science University. All participants were provided and signed written informed consent prior to participation.

### Subjects and Clinical Assessments

Participants were recruited from an ongoing Pacific Udall Center project at two sites: the University of Washington/Veterans Affairs Puget Sound Health Care System and Oregon Health Sciences University/Veterans Affairs Portland Health Care System. Each participant was screened for TMS eligibility before enrollment. Inclusion criteria included diagnosis of idiopathic PD using the United Kingdom Parkinson's Disease Society Brain Bank (UKBB) criteria (18) and ability to stand unsupported for 30 s. Exclusion criteria were inability to walk for 2 min without an assistive device, any TMS contraindication, any musculoskeletal injury/abnormality that would affect mobility, or any neurodegenerative disorder aside from PD. All participants with PD were tested “ON” dopaminergic medications due to the interaction with SAI (19), and no participants on cholinergic medication was recruited to the TMS portion of the Pacific Udall Center project.

The Movement Disorders Society Unified Parkinson's Disease Rating Scale (MDS-UPDRS) part III, the modified Hoehn & Yahr (H&Y) score, and the Montreal Cognitive Assessment (MoCA) were used to assess motor severity and global cognition, respectively. A consensus committee comprised of movement disorder neurologists and a neuropsychologist reviewed data from each participant to determine if UKBB criteria were satisfied and to assign a cognitive diagnosis (normal, mild cognitive impairment, or dementia) (20).

### Genotyping

Genomic DNA was extracted from peripheral blood or saliva samples using standard procedures. The entire *GBA* coding region was screened using Sanger sequencing to capture all known pathogenic mutations (defined as those reported in patients with Gaucher disease) and the E326K polymorphism (rs2230288). *APOE* rs429358 and rs7412, which define the ε2, ε3, and ε4 alleles, were genotyped using TaqMan assays. All sequencing and genotyping was performed at a single laboratory in Seattle implementing methods previously described (8, 21).

### Transcranial Magnetic Stimulation

TMS of the motor cortex was performed with a Magstim 200 (Magstim Co.). A figure-of-eight coil (external loop diameter of 9 or 7 cm, site specific) was positioned over the hemisphere associated with the most affected side in PD participants and the dominant side in control participants. Motor-evoked potentials (MEPs) were recorded from the first dorsal interosseous muscle through disposable, Ag/AgCl surface electrodes. Samples were amplified (gain: 2000) and bandpass filtered (100 Hz−5 kHz) using BIOPAC MP150 system (BIOPAC Systems, Inc) or amplified (CED 1902 isolated preamplifier, Cambridge Electronics), converted from analog to digital (Sampling rate 40 KHz, PowerLab, ADInstruments), and recorded for offline analysis (LabChart, ADInstruments). Resting motor threshold was determined as the percentage of the minimum stimulator output to elicit an MEP of 50 μV in five out of 10 trials.

### Short Latency Afferent Inhibition (SAI)

SAI was performed using a modified version of a protocol previously described (22). A peripheral, electric conditioning stimulus was applied over the median nerve followed by the central test stimulus, TMS. The intensity of the conditioning stimulus was set at the amplitude required to elicit a visible twitch of the thenar muscles. The N20 latency was not individualized by somatosensory evoked potentials, instead, the time of 20 ms for the N20 was used across all participants. The interstimulus intervals (ISI) from N20 + 0 ms to N20 + 5 ms were applied in a randomized block format. Five unconditioned trials were collected within each block of conditioned trials, in a pseudo-random order, for a total of 30 unconditioned trials. A total of 10 trials were collected for each condition and the conditioned peak-to-peak MEP magnitudes averaged for each ISI. There was a minimum of seven seconds between each trial. A grand mean of the ISIs is expressed as the percentage of the unconditioned MEP magnitudes. Participants were instructed to remain at rest, while sitting as still as possible, and refrain from keeping their eyes closed.

### Gait and Postural Sway

Inertial sensors (Opals, APDM Inc.) were placed on each wrist and foot, around the waist, and over the sternum to record mobility measures (23). Gait was characterized over a 2-min walk, over a 7-meter path, requiring 180° turns at the ends of the marked path. Comprehensive measures of gait and balance were combined into four domains based on our previous principle component analysis using a larger cohort, which included people with PD in this study (10). The four domains of gait were pace/turning, rhythm, variability, and trunk movement. Postural sway was characterized while participants stood quietly for 1 min looking straight ahead with feet width standardized by a template (24). Similar to the gait variables, sway variables of interest were divided into two domains, sway area/jerkiness and sway velocity.

### Statistical Analyses

Data were inspected for normality using histograms and the Kolmogorov-Smirnov test of normality. All non-normally distributed data were log-base 10 transformed. Domain scores were calculated by averaging the Z-scores for each gait and sway variable. Z-scores were multiplied by −1 to reverse scaling if needed for consistent sign in domain score calculations. A one-way ANOVA compared PD vs. control group differences for demographic information and SAI, with *post hoc* analyses Tukey corrected for multiple comparisons. A Chi-Squared test assessed the sex differences among the groups. Two-by-two general linear models (GLM) were used to assess the effects of PD status and *APOE* ε4 carrier status on SAI, gait domains, and sway domains, controlling for age, sex, and collection site. The *GBA* group was excluded from this GLM model. To determine if GBA variants affected SAI and gait/postural sway within the PD cohort, we used a separate GLM, controlling for age, sex, and collection site. Neither of the OA groups were included in this GLM. Participants who carried a pathogenic mutation or the E326K polymorphism where combined into a single *GBA* group. Alpha was set *a priori* to *p* < 0.05. IBM SPSS version 25 was used for statistical analyses.

## Results

Ninety-three participants with idiopathic PD (53 non-carriers; 23 ε4 carriers; 17 *GBA* variants) and 72 healthy older adults (OA; 45 non-carriers; 27 ε4 carriers) participated (Table 1). The consensus committee assigned 50 participants with MCI and nine participants with dementia in the PD, while 23 participants in the OA group were assigned with MCI and one with dementia. 13/50 PD MCI and 2/9 PD dementia were ε4 carriers, while 9/50 PD MCI and 3/9 PD dementia were *GBA* variants. 10/23 OA MCI and the one OA with dementia were ε4 carriers. Age was a significant factor [F_(1, 166)_ = 5.75; *p* = 0.02], and *post hoc* analyses showed the PD *GBA* group was significantly younger than the rest of the groups (all *p*-values ≤ 0.03). Only the MDS-UPDRS III was significantly different between the PD [24.2 (12.7)] and OA [1.9 (2.9)] groups [F_(1, 160)_ = 211.98; *p* < 0.001]. *Post hoc* analyses reveled the OA genetic groups were not different from each other, but both OA groups had lower MDS-UPDRS III than all the PD groups (all *p*-values < 0.001).

**Table 1:** Demographic and clinical information.

	**OA**		**PD**			**OA vs. PD**
	**Non-Carrier**	**ε4**	**Non-Carrier**	**ε4**	**GBA**	***p*-value**
*n*	45	27	53	23	17	N/A
Gender (F/M)	25/20	15/12	14/39	9/14	3/14	<0.001
Age (yrs)	69.9 (6.1)	71.4 (7.3)	68.7 (6.7)	67.5 (8.9)	62.5 (8.2)	=0.018
MoCA Score	26.7 (2.6)	25.5 (3.6)	25.9 (3.2)	26.4 (2.3)	26.1 (2.7)	=0.612
Modified H&Y			2.0 (1–4)	2.0 (2–3)	2.0 (1–3)	N/A
MDS-UPDRS III	1.5 (2.0)	2.7 (4.2)	23.9 (12.5)	25.2 (12.7)	23.8 (13.9)	<0.001
Disease Duration (yrs)			8.2 (5.2)	7.1 (4.1)	7.4 (3.3)	N/A
LEDD			694.4 (481.0)	824.3 (526.4)	771.8 (656.9)	N/A

*Mean (standard deviation). H&Y presented as median (min-max). The modified H&Y and MDS-UPDRS III scores were significantly difference between PD and OAs, but not within the PD or OA groups. Neither disease duration nor LEDD were significantly different among the PD genetic subgroups*.

### Short-Latency Afferent Inhibition (SAI)

**Figure 1:**
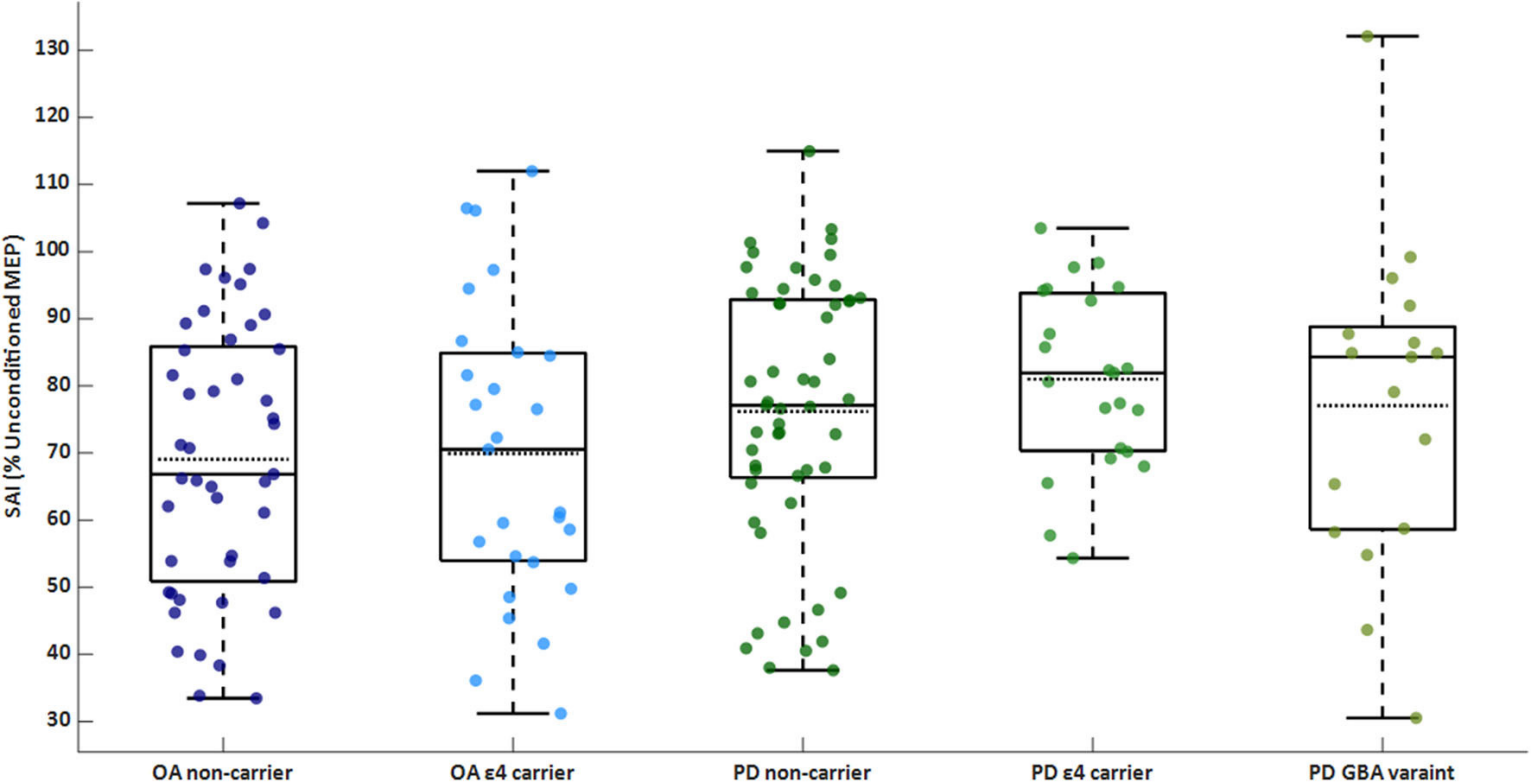
Box/scatter plot of sensorimotor inhibition (SAI) for each group. A higher percentage is associated with less (i.e., worse) inhibition. Dashed lines represent group means. SAI is significantly different between the PD and OA groups (*p* = 0.004). OA, older adults; PD, Parkinson's disease.

The PD group exhibited worse sensorimotor inhibition than the control group [PD: 77.6 (18.2)% inhibition; OA: 69.4 (20.8)% inhibition; F_(1, 157)_ = 8.31]. There was no significant main effect of ε4 carrier status or interaction between PD status and genetics on SAI. Within the PD group, there was no significant main effect of genetics for SAI. SAI for each genetic subgroup of OA and PD are shown in Figure 1.

### Gait Domains

The main effect of PD status was significant [F_(4, 138)_ = 7.49; *p* < 0.001] for the pace/turning domain [F_(1, 141)_ = 22.61; *p* < 0.001] and variability domain [F_(1, 141)_ = 10.05; *p* = 0.002]. Specifically, gait was slower and more variable in the PD than the control group. The main effect for ε4 carrier status on gait domains was not significant. Further, there was no main effect for genetic status within the PD group. The gait domain scores are presented in Table 2. Individual gait characteristics that comprise each gait domain are provided in Table 3 to provide greater detail about gait performance.

**Table 2:** Gait and postural sway domain scores.

		**OA**		**PD**			**OA vs. PD**
		**Non-carrier**	**ε4**	**Non-carrier**	**ε4**	**GBA**	***p*-value**
Gait Domains	Pace/Turning	0.21 (0.63)	0.33 (0.64)	−0.19 (0.86)	−0.31 (0.90)	−0.37 (0.83)	<0.001
	Rhythm	−0.07 (0.90)	−0.07 (0.80)	0.05 (0.79)	−0.11 (0.96)	0.09 (1.04)	=0.829
	Variability	−0.28 (0.60)	−0.22 (0.62)	0.15 (1.00)	0.08 (0.80)	0.43 (0.97)	=0.002
	Trunk Movement	0.05 (0.33)	−0.06 (0.32)	0.04 (0.45)	−0.16 (0.35)	0.06 (0.62)	=0.986
Postural Sway Domains	Area/Jerkiness	−0.38 (0.53)	−0.44 (0.54)	0.31 (1.03)	0.18 (0.82)	0.62 (1.08)	<0.001
	Velocity	−0.27 (0.59)	−0.31 (0.75)	0.23 (0.85)	0.16 (0.95)	0.42 (0.82)	<0.001

*Mean (standard deviation)*.

**Table 3:** Individual gait characteristics.

		**OA**		**PD**			
		**Non-carrier**	**ε4**	**Non-carrier**	**ε4**	**GBA**	
Gait	Pace/Turning	Gait Speed (m/s)	1.1 (0.2)	1.1 (0.2)	1.1 (0.2)	1.0 (0.2)	1.0 (0.2)
		Stride Length (m)	1.2 (0.1)	1.2 (0.2)	1.2 (0.2)	1.1 (0.2)	1.1 (0.2)
		Foot Strike Angle (°)	23.1 (4.0)	23.3 (4.6)	19.7 (6.3)	18.4 (6.1)	19.2 (6.4)
		Turns Duration (s)	2.2 (0.3)	2.1 (0.3)	2.4 (0.4)	2.4 (0.4)	2.5 (0.4)
		Peak Turn Velocity (°/s)	180.9 (33.7)	186.3 (43.4)	160.6 (42.3)	166.4 (30.5)	151.2 (29.8)
		Steps In Turn (count)	3.8 (0.6)	3.6 (0.4)	4.1 (0.8)	4.1 (0.8)	4.1 (0.8)
	Rhythm	Stride Time (s)	1.1 (0.1)	1.1 (0.1)	1.1 (0.1)	1.1 (0.1)	1.1 (0.1)
		Stance Time (% gait cycle)	60.4 (1.8)	60.3 (1. 6)	60.6 (1.7)	60.3 (1.9)	60.6 (2.1)
		Swing Time (% gait cycle)	39.6 (1.8)	39.7 (1.6)	39.4 (1.7)	39.7 (1.9)	39.4 (2.1)
	Variability	Foot Strike Angle Variability	2.08 (0.62)	2.21 (0.73)	2.43 (1.19)	2.35 (0.73)	2.62 (0.79)
		Stride Time Variability	0.02 (0.01)	0.03 (0.01)	0.03 (0.01)	0.03 (0.01)	0.03 (0.01)
		Stance Time Variability	0.77 (0.18)	0.78 (0.20)	0.95 (0.39)	0.90 (0.30)	1.02 (0.34)
		Swing Time Variability	0.77 (0.18)	0.78 (0.20)	0.95 (0.39)	0.90 (0.30)	1.02 (0.34)
		Stride Length Variability	0.04 (0.02)	0.04 (0.02)	0.05 (0.02)	0.05 (0.02)	0.06 (0.03)
	Trunk Movement	Trunk Coronal RoM (°)	5.2 (2.1)	4.6 (1.3)	5.1 (2.0)	4.4 (1.6)	5.0 (3.4)
		Trunk Sagittal RoM (°)	4.1 (0.9)	4.3 (0.8)	4.6 (1.5)	4.0 (1.0)	4.3 (1.1)
		Trunk Transverse RoM (°)	8.6 (2.8)	10.3 (3.3)	10.3 (3.7)	10.1 (4.1)	8.8 (2.7)
Postural Sway	Area/Jerkiness	Sway Area (m^2^/s^5^)	0.003 (0.002)	0.003 (0.002)	0.009 (0.012)	0.006 (0.006)	0.014 (0.025)
		Jerk AP (m^2^/s^5^)	0.002 (0.002)	0.002 (0.002)	0.004 (0.005)	0.003 (0.003)	0.007 (0.010)
		Jerk ML (m^2^/s^5^)	0.001 (0.0004)	0.0004 (0.0002)	0.003 (0.013)	0.001 (0.002)	0.008 (0.025)
		RMS AP (m^2^/s)	0.071 (0.025)	0.070 (0.026)	0.106 (0.066)	0.091 (0.049)	0.110 (0.042)
		RMS ML (m^2^/s)	0.021 (0.009)	0.022 (0.011)	0.043 (0.041)	0.042 (0.032)	0.052 (0.045)
	Velocity	Velocity AP (m/s)	0.253 (0.200)	0.235 (0.154)	0.403 (0.395)	0.356 (0.384)	0.398 (0.258)
		Velocity ML (m/s)	0.077 (0.052)	0.092 (0.080)	0.165 (0.221)	0.177 (0.153)	0.217 (0.257)

*Mean (standard deviation). All variability measures are presented as standard deviations*.

### Sway Domains

Similar to the gait results, the main effect for PD status was significant [F_(2, 140)_ = 12.18; *p* < 0.001]. The PD group showed larger sway area/jerkiness [F_(1, 141)_ = 24.22; *p* < 0.001] and larger sway velocity [F_(1, 141)_ = 13.08; *p* < 0.001] compared to the OA group. There was no main effect for ε4 carrier status on the sway domains. The sway domain scores are presented in Table 2. No significant main effect for genetic status within the PD group was found for any of the sway domains. Individual postural sway characteristics that comprise each gait domain are provided in Table 3 to provide greater detail about postural sway performance.

### Relationship Between SAI and Mobility Domains

**Figure 2:**
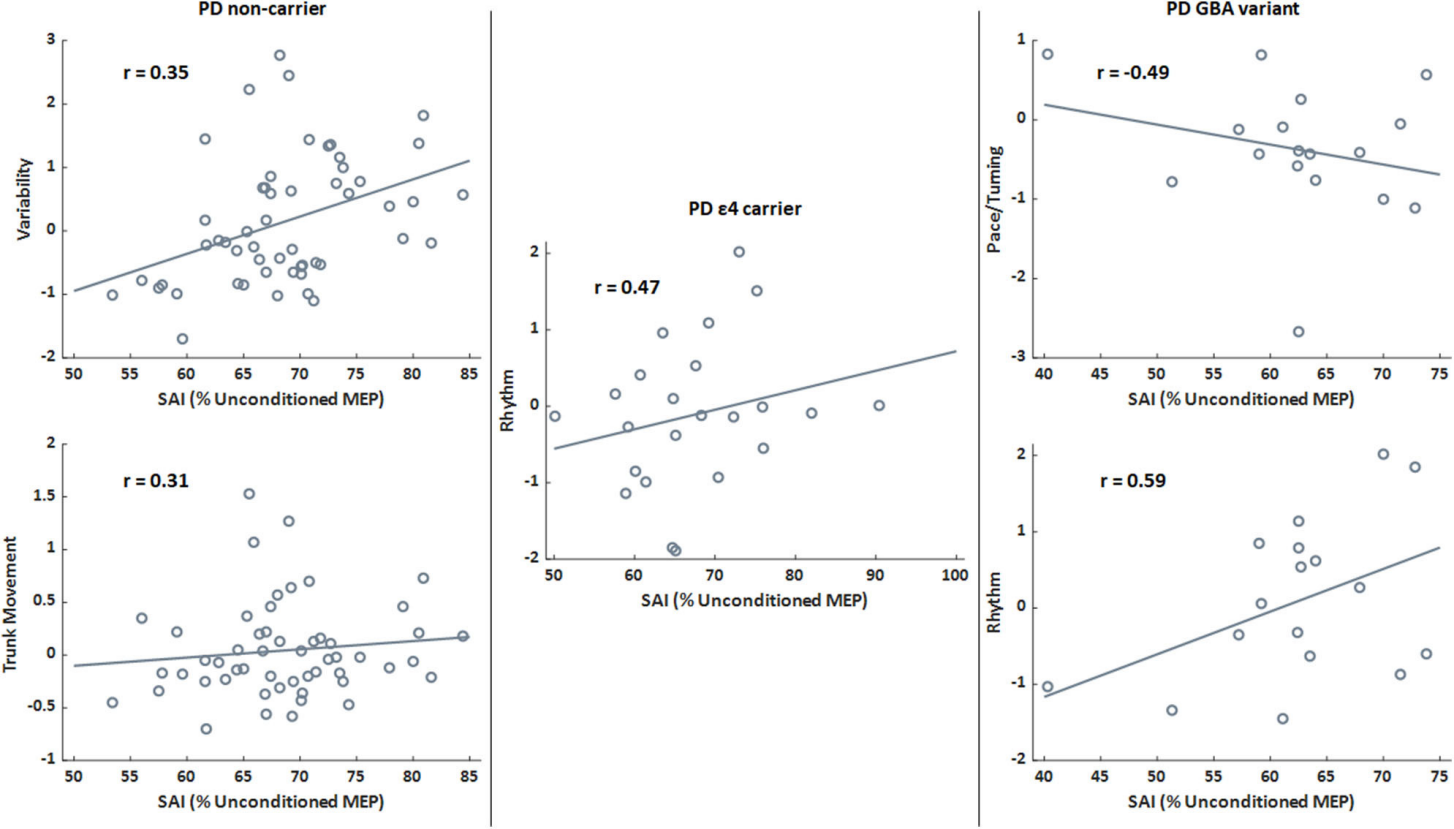
Scatter plots presenting the relationships between sensorimotor inhibition (SAI) and gait domains for the PD genetic subgroups only. Lines indicate the best fit line. A higher percentage (SAI) is associated with less (i.e., worse) inhibition. Higher domain scores indicate faster pace/turning, increased rhythm (increased times for stride, stance, and swing phases), and greater variability. *r* is the Pearson's correlation coefficient for the relationship between SAI and relevant gait domain score. All correlations presented are significant (*p* < 0.05).

SAI significantly correlated with gait variability (Pearson's *r* = 0.27; *p* = 0.017) and trunk movement (Pearson's *r* = 0.23; *p* = 0.045) in the PD group. Though mobility performance was not different genetic groups in The PD cohort, the relationships between SAI and variability (Pearson's *r* = 0.35; *p* = 0.010) and between SAI and trunk movement (Pearson's *r* = 0.31; *p* = 0.025) only remained for the PD non-carriers. SAI only related to rhythm (Pearson's *r* = 0.47; *p* = 0.024) for the PD ε4 group, while SAI related to pace/turning (Pearson's *r* = −0.49; *p* = 0.046) and rhythm (Pearson's *r* = 0.59; *p* = 0.013) in the PD *GBA* group. There was no relationship between SAI and postural sway domains in the PD group. Scatter plots for significant relationships within the PD are in Figure 2.

SAI was significantly correlated with the gait pace/turning domain (Pearson's *r* = −0.24; *p* = 0.046) in the OA group. Genetic subgroup analyses revealed no relationships for the OA group. There was no relationship between SAI and postural sway domains for the OA group.

## Discussion

This study investigated how genetic factors influence sensorimotor inhibition and mobility in OAs and people with PD. Although people with PD had worse SAI, gait, and balance than OAs, our results suggest that carriers of *APOE* ε4 allele or *GBA* variants do not show worse SAI, nor worse gait and postural sway than non-carriers. However, worse sensorimotor inhibition was associated with worse gait (but not standing postural sway) in all genetic subgroups of PD, but not OA groups. In fact, heterogeneity of specific domains of gait most affected in people with PD (decreased pace/turning, increased variability, and increased rhythm) depended upon the genetic status of the PD group. Thus, cortical sensorimotor inhibition appears more critical for gait than for standing balance and more critical for people with PD than for older adults without PD.

We previously reported (Martini et al., under review at J Gerontol: Med Sci) that people with PD had significant mobility dysfunction compared to healthy OAs, including slower gait speed/turn velocity and greater gait variability, postural sway area/jerkiness, and sway velocity, consistent with previous investigations (25–27). Our previous report included 81 of the 93 people with PD and 69 of the 72 OAs in this report (Martini et al., under review at J Gerontol: Med Sci). The current study used mobility domain scores, based on principal component analysis (10), rather than individual metrics of postural sway and gait, which can lead to inflated, type-one errors.

Genetic status was not a significant factor for gait or sway performance in people with idiopathic PD, contrary to our hypothesis. *GBA* is a strong risk factor for PD and particularly PD with more severe cognitive and gait and balance decline than PD without *GBA* (8, 9, 17). Since ACh is known to be important for cognition (28) and cognition is known to be related to gait speed in both older adults and people with PD (1, 29), we expected people with PD who had *GBA* variants to show worse SAI as well as worse balance and gait than non-carriers A larger cohort of people with PD and *GBA* variants (*n* = 58) from our PUC investigation reported that people with PD and GBA a variant had a quicker progression through motor and cognitive decline than non—*GBA* variant PD (17). Our subset from that sample of people with GBA variants is smaller, with milder parkinsonian symptoms based on the MDS-UPDRS III (17).

Like *GBA, APOE* ε4 allele status was not associated with worse mobility. The *APOE* ε4 allele is not a risk factor for PD, but it is a risk factor for dementia. Since decline in cognition is associated with decline in balance control with aging and with PD (30, 31), we hypothesized that people with PD who are carriers of *APOE* ε4 allele would have more loss of ACh than non-carriers and therefore show worse SAI and worse balance and gait than non-carriers. In fact, *APOE* ε4 allele carriers with PD have been shown to have worse executive function than non-carriers and executive function has been associated with slower gait speed (32) in otherwise healthy OAs and with increased variability (33) and Alzheimer's disease. However, we found no evidence that *APOE* ε4 status in people with PD was associated with worse SAI nor worse balance or gait. Though, the MoCA scores for the genetic subsets were not statistically different and were above the cognitive impairment cut off score of 23, which was reported to have higher sensitivity and specificity than higher cut off scores (34). Longitudinal studies, which incorporate a comprehensive cognitive batteries, are needed to determine if PD *APOE* ε4 carriers would show faster progression of their balance and gait disorders over time, similar to older men without PD (11).

Genetic status was not a significant factor for gait or sway performance in healthy OAs. This contradicts previous reports that observed smaller stride length in male OA ε4 allele carriers than non-carriers (35). Longitudinal analyses reported similar observations, with an accelerated decline in gait speed and increase in gait variability in ε4 allele carriers over 1 year (11, 36). The accelerated decline in gait speed was only noted for OA males (11), which suggests that there may be a sex influence on ε4 allele carrier status and increased risk of gait dysfunction over time. Despite the ε4 allele being linked to an increased risk for dementia (37), a separate study found no change in MoCA scores over a year (36), suggesting an increased motor decline not directly linked to cognitive decline. Thus, assessing changes in gait and balance performance across time may be a better indicator of mobility dysfunction in male ε4 allele carriers than a cross-sectional approach.

Consistent with previous investigations, we show that people with PD do not inhibit the motor cortex as much with a sensory afferent signal compared to OAs (1, 2, 38) (Martini et al., under review at J Gerontol: Med Sci). *GBA* and ε4 allele status did not affect SAI, similar to leucine-rich repeat kinase 2 mutation nor a *Parkin* gene mutation (5–7). Sensorimotor inhibition, as measured with SAI, is thought to reflect levels of cortical ACh neurotransmitter activity (3), The basis for the notion that SAI is mediated by cholinergic activity is that SAI is impaired in Alzheimer's disease, a disorder known to result in loss of ACh, and because medications that increase ACh also improve the SAI (13, 39–41). Recent theories of the neural pathways responsible for SAI suggests that the subthalamic nucleus or paramedian thalamic nuclei are responsible for inhibition of the primary motor cortex either directly, or indirectly, via the primary sensory cortex (38, 42). The theoretical pathway that incorporates the subthalamic nucleus was posited as a result of the modulatory effects of deep brain stimulation on the subthalamic nucleus in human and animal studies (38). The theoretical pathway, involving the paramedian thalamic nuclei is based off of the connections between SAI and cholinergic pathways as observed in drug and disease studies (13, 41, 43). Importantly, dopamine replacement therapy is linked to worse SAI in people with PD (19), suggesting that cholinergic activity is not the only influence on the inhibitory response recorded in SAI. A reciprocal circuit among the basal ganglia (e.g., subthalamic nucleus), thalamus, and cortex could be the primary neural pathway responsible for SAI, as a measure of sensorimotor inhibition (38, 42, 44). Although the specific neural mechanisms underlying the SAI are a debated, a recent review of the topic details the complex neural pathways between the basal ganglia and motor cortices, moderated by thalamus that may be involved (44).

The effect of PD status on SAI suggests that the pathway between the substantia nigra pars compacta, ventral thalamus, and motor regions are responsible for the inhibitory response observed. The ventral lateral thalamic nuclei receive input from the basal ganglia, the substantia nigra in particular, as well as proprioceptive input (44). The substantia nigra degeneration with concomitant loss of dopamine neurotransmitter is responsible for Parkinsonism but dopamine replacement therapy does not alleviate all gait and balance impairments in PD, and may even worsen some domains of mobility (45–47). In addition, proprioception is a critical sensory input for control of gait and balance and is known to be impaired in PD (48).

In this same cohort of people with PD, we previously reported that sensorimotor inhibition was specifically related to stride length variability, foot strike angle variability, and jerkiness of sway (Martini et al., under review at J Gerontol: Med Sci). The relationship between SAI and gait domains, but not with balance domains, observed here suggests that sensorimotor inhibition is more critical for control of dynamic balance, than static, standing balance, which may be more controlled by thalamic and brainstem mechanisms (49). The relationship between worse sensorimotor increased gait variability could be an indicator of decreased gait automaticity, with increased reliance on frontal cortical areas (50, 51). Indeed, increased frontal cortex activity and gait variability is found in people with PD and freezing of gait (52). Ultimately, loss of gait automaticity is related to an increased risk of falls across gait disorders (53).

SAI was differently related to gait domains across the genetic subgroups in the PD group. Worse SAI was related to increased gait variability and trunk movement in the PD non-carrier group, which may reflect impaired dynamic balance as postural stepping responses to correct lateral body center of mass displacements interrupt steady state rhythmic gait (47, 54, 55). SAI was significantly related to increased rhythm in the PD ε4 group. Increased rhythm, which is derived from stride time, stance time, and swing time, suggests that the PD ε4 group has longer stride and stance times when sensorimotor inhibition is worse. Lastly, SAI related to decreased pace/turning and increased rhythm in the PD *GBA* variant group. This relationship may permit SAI to be a marker of slowed gait performance due to bradykinesia or an early adoption of a conservative gait strategy, in an attempt to prevent falls.

SAI was associated with the gait pace/turning domain in older adults, but there were no effects of the ε4 allele on gait or the relationship between sensorimotor inhibition and gait in the OA group. Previous investigations found no difference in sensorimotor inhibition between otherwise healthy young and OA groups (56, 57). However, both sensorimotor inhibition and gait speed were reduced in OA fallers compared to OA non-fallers (2). Adopting a slower gait pace may be a compensatory mechanism for poorer sensorimotor inhibition as a means for preventing falls. These results suggest that OAs with low sensorimotor inhibition may need an intervention to help reduce potential falls. SAI may be a quick, non-invasive assessment for identifying OAs who could benefit from an intervention. Though, the appropriate type of intervention would depend on the neurological mechanism responsible for SAI.

There are potential limitations to the interpretation of the results of this investigation. TMS was collected at two different academic centers. However, we controlled for this statistically and the SAI is calculated as a percentage of each participant's own MEPs, mitigating device or administrator influence. Further, we created a site variable and used it as a covariate in analyses. The effects of sex on SAI are not fully understood, so we adjusted for sex statistically. The sample sizes for the ε4 allele carriers and the *GBA* carriers were small. We attempted to mitigate this by analyzing the ε4 allele carriers and non-carriers across PD status, and ε4 allele and *GBA* variant status within the PD group only. *GBA* variants only exist in 8% of the idiopathic PD population, which makes this population difficult to recruit. The inclusion criteria requiring participants to stand for at least 30 s unassisted could have biased the sample away from more severely affected ε4 allele carriers and *GBA* carriers. Using the first dorsal interosseous muscle to measure SAI, opposed to a lower-limb muscle, when attempting to elucidate relationships between SAI and gait/sway characteristics is a limitation that may affect the generalizability of the results. However, previous groups implemented the same SAI methodology when relating SAI to gait performance (1, 2, 4).

Among people with PD, *APOE* and *GBA* genetic status did not affect either sensorimotor inhibition or measures of gait and postural sway. However, dependent on genetic status, worse sensorimotor inhibition was related to increased gait variability, impaired temporal rhythm and/or slower gait pace/turning in this PD group. This dichotomy of the relationships between sensorimotor inhibition and gait domains for people with PD with different genetic status could be related to the dependence of each group on cortical control of different aspects of gait. Longitudinal studies could elucidate more robust relationships between sensorimotor inhibition and mobility, while factoring in the role of cognition, in the ε4 and *GBA* variant subgroups of people with PD.

## Data Availability Statement

The raw data supporting the conclusions of this article will be made available by the authors, without undue reservation.

## Ethics Statement

The studies involving human participants were reviewed and approved by This study was approved by Institutional Review Boards at both University of Washington and Oregon Health & Science University. The patients/participants provided their written informed consent to participate in this study.

## Author Contributions

DM: research organization and execution, statistical design and execution, and writing first draft. RM: research organization and execution, and statistical design, and draft review and critique. VK, AH, KC, S-CH, and IM: research organization and execution, and draft review and critique. CZ: research conception, organization and execution and draft review and critique. JO: research organization and execution and draft review. KP: statistical critique and draft review and critique. KE: research conception, organization, statistical critique, and draft review and critique. JL: research conception, statistical critique, and draft review and critique. TG: research organization and draft review and critique. TM, JQ, and FH: research conception, organization and execution, statistical critique, and draft review and critique. All authors contributed to the article and approved the submitted version.

## Conflict of Interest

FH has a significant financial interest in APDM, a company that may have a commercial interest in the results of this research and technology. This potential conflict has been reviewed and managed by OHSU. The remaining authors declare that the research was conducted in the absence of any commercial or financial relationships that could be construed as a potential conflict of interest.
